# Association between video game addiction, stress, and bruxism in adolescents: a cross-sectional study

**DOI:** 10.1186/s12903-025-06568-0

**Published:** 2025-07-15

**Authors:** Ceylan Güzel, Fatma Dilek Erten, Ayda Seyidoğlu, Sümer Münevveroğlu

**Affiliations:** 1 Department of Oral and Maxillofacial Surgery, Faculty of Dentistry, Istanbul Medipol University, Istanbul, Türkiye; 2 Department of Oral and Maxillofacial Surgery, Faculty of Dentistry, Graduate School of Health Sciences, Istanbul Medipol University, Istanbul, Türkiye

**Keywords:** Awake Bruxism, Sleep Bruxism, Stress levels, Video game addiction

## Abstract

**Background:**

This study aims to examine the association between video game addiction stress levels, and both awake and sleep bruxism in adolescents. Given the increasing prevalence of digital addiction among young populations, understanding its potential impact on oral health is essential.

**Methods:**

A cross-sectional study was conducted with 300 adolescents aged 10–19 years. Participants completed an online survey assessing video game addiction using the short form of the Digital Game Addiction Scale − 21 (DGAS-21), stress levels using the Perceived Stress Scale (PSS), and bruxism through self-reported questionnaires based on the American Academy of Sleep Medicine (AASM) criteria. Statistical analyses included chi-square tests, independent t-tests, and point-biserial correlation analysis. A significance level of *p* ≤ 0.05 was considered statistically significant.

**Results:**

The prevalence of video game addiction in the sample was 50.3% (*n* = 151), with males significantly more affected than females (*p* < 0.05). Participants with video game addiction exhibited higher stress levels compared to non-addicted individuals (*p* < 0.001). A statistically significant association was found between video game addiction and both awake bruxism (*r* = 0.31, *p* < 0.001) and sleep bruxism (*r* = 0.28, *p* < 0.001). However, stress levels were not significantly correlated with bruxism (*p* > 0.05), suggesting that factors beyond stress may contribute to this relationship.

**Conclusion:**

These findings indicate that video game addiction is associated with an increased likelihood of both awake and sleep bruxism in adolescents. However, the nature of this relationship remains unclear. Further longitudinal and experimental studies incorporating objective bruxism assessments are necessary to clarify potential causal mechanisms. Preventive measures focusing on digital well-being and stress management strategies should be considered in adolescent healthcare interventions.

**Supplementary Information:**

The online version contains supplementary material available at 10.1186/s12903-025-06568-0.

## Background

Bruxism is defined as a repetitive activity of the masticatory muscles, characterized by clenching or grinding of the teeth, and can occur during both wakefulness (awake bruxism) and sleep (sleep bruxism) [1, 2]. Recent consensus guidelines emphasize that bruxism should no longer be classified as a mere parafunctional habit but rather as a complex, multifaceted condition that can serve both protective and potentially harmful roles depending on its intensity and frequency [3]. Although bruxism has been associated with jaw pain, headaches, and temporomandibular joint (TMJ) dysfunction in some individuals, it is not necessarily a pathological condition nor exclusively linked to TMJ disorders [1, 3].

The etiology of bruxism is considered multifactorial, involving interactions between neurological, genetic, psychological, and behavioral factors [4, 5]. Among these, psychological stress has been frequently studied as a potential contributor to bruxism, although findings remain inconsistent across different populations [6, 7]. While some studies suggest that stress increases the frequency and intensity of bruxism episodes, others indicate that additional mediating factors may be involved [8].

In recent years, video game addiction has emerged as a growing concern, particularly among adolescents, with potential implications for both mental and physical health [9, 10]. Defined as a compulsive pattern of gaming that leads to impaired daily functioning, video game addiction has been linked to increased stress, anxiety, and sleep disturbances [11–13]. Evidence suggests that problematic gaming behavior is positively associated with perceived stress levels and emotional dysregulation in adolescents [12–14].

Given the neuromuscular tension and heightened cognitive engagement associated with prolonged gaming, there is growing speculation that excessive video game use may contribute to bruxism, particularly in younger populations.

Although previous studies have examined the association between stress and bruxism, as well as the impact of video game addiction on stress, limited research has investigated whether video game addiction itself is an independent risk factor for bruxism in adolescents. Furthermore, while some studies have explored bruxism in children and younger age groups [14], there is a lack of data specifically addressing how gaming-related factors may contribute to bruxism in adolescent populations.

This study aims to examine the relationship between video game addiction, stress levels, and both awake and sleep bruxism in adolescents aged 10–19 years. Unlike prior studies that have focused primarily on stress as a mediating factor for bruxism, this investigation seeks to determine whether video game addiction itself is directly associated with bruxism, independent of stress levels. Additionally, we explore potential gender differences in these associations, addressing an underexamined aspect of gaming-related health effects.

We hypothesize that adolescents with video game addiction will exhibit higher rates of awake and sleep bruxism, regardless of their perceived stress levels.

By clarifying whether video game addiction contributes to bruxism beyond the effects of stress, this study provides new insights into the potential oral health consequences of digital addiction in adolescents.

## Materials and methods

This cross-sectional study was conducted to investigate the relationship between video game addiction, psychological stress, and bruxism (both awake and sleep) in adolescents aged 10–19 years. The study was approved by the Ethics Committee of Istanbul Medipol University, Clinical Research Ethics (Decision No: E-10840098-772.02-4359, Date: 01/08/2022) and adhered to the principles outlined in the Declaration of Helsinki. Written informed consent was obtained from all participants prior to their inclusion in the study. For participants under the age of 18, parental consent was also obtained.

A power analysis was conducted to determine the minimum sample size required for the study. Based on an expected medium effect size (Cohen’s d = 0.5), a significance level of α = 0.05, and a power of 0.80, the analysis indicated that a minimum of 279 participants would be required. To account for potential exclusions and incomplete responses, 346 adolescents aged 10–19 years were initially invited to participate in the study. Participants were recruited from secondary and high schools and in Istanbul/Türkiye, using a convenience sampling approach.

Adolescents aged 10–19 years who provide informed consent, with parental consent required for minors, were included in the study. Participants must have no history of drug or substance addiction, no current use of psychiatric medications, and no prior treatment for TMJ disorders.Individuals were excluded if they provided incomplete or inconsistent survey responses, had a self-reported history of neurological or psychiatric disorders, or were using medications that could affect muscle activity or stress levels, such as muscle relaxants or antidepressants.

After applying the inclusion and exclusion criteria, 300 participants were included in the final analysis.

The survey was administered online using Google Forms and consisted of four sections: demographic information, bruxism assessment, stress measurement, and video game addiction evaluation. The survey was designed to be completed in approximately 15–20 min. Original surveys were conducted in Turkish. Original versions and English translations are provided in Supplementary Materials.

### Data collection

The survey was distributed to participants via social media platforms. The first page of the survey provided detailed information about the study objectives, procedures, and confidentiality measures. Participants were informed that their participation was voluntary and that they could withdraw at any time without penalty. To ensure data quality, participants were required to answer all questions before submitting the survey. Incomplete responses were excluded from the analysis.Age, gender, and medication use were collected to characterize the study population.

Sleep bruxism was evaluated through a questionnaire based on the diagnostic criteria of the American Academy of Sleep Medicine (AASM) [2, 15], consisting of three sections. The first section assessed self-reported or partner-reported teeth grinding or clenching during sleep, while the second section evaluated self-awareness of these activities upon awakening. The third section examined the presence of associated symptoms, including jaw pain, headaches, or tooth wear. A diagnosis of sleep bruxism was established when a participant responded affirmatively to at least one of the first two sections and reported at least one symptom from the third section.

The presence of awake bruxism was determined using a single-question assessment in which participants were asked whether they had noticed themselves grinding or clenching their teeth during the day in the past six months. Responses were recorded as either “yes” or “no.”

In accordance with recent diagnostic criteria, only clenching and grinding behaviors were considered in the assessment of bruxism. Bracing and thrusting behaviors, although part of the extended definition of bruxism, were not evaluated in this study.

Stress levels were measured using the 10-item Perceived Stress Scale (PSS), a validated self-report tool that assesses the degree to which individuals perceive their lives as stressful over the past four weeks [16]. Responses were recorded on a 5-point Likert scale ranging from “never” (0) to “very often” (4). Total scores ranged from 0 to 40, with higher scores indicating greater perceived stress. The Turkish version of the PSS has been validated and demonstrated high reliability (Cronbach’s α = 0.85) [16, 17].

Video game addiction was assessed using the short form of the DGAS-21, developed by Lemmens et al. [18]. This 21-item scale evaluates problematic gaming behaviors across seven subscales: salience, tolerance, mood modification, relapse, withdrawal, conflict, and problems. Responses were recorded on a 5-point Likert scale ranging from “never” (1) to “very often” (5). A total score of ≥ 50 was used to classify participants as having video game addiction, based on established cut-off points.

### Statistical analysis

Data were analyzed using IBM SPSS Statistics 22 software (IBM SPSS, Turkey). Descriptive statistics summarized demographic characteristics, prevalence rates, and scale scores, with continuous variables reported as means ± standard deviations (SD) and categorical variables as frequencies and percentages. Chi-square tests examined differences in categorical variables, while independent t-tests compared means between groups. Point-biserial correlation analysis assessed relationships between video game addiction (a dichotomous variable) and continuous variables. The significance level was set at *p* ≤ 0.05. To ensure validity and reliability, only well-established and validated scales were used, including the previously validated Turkish versions of the PSS and DGAS-21. The bruxism questionnaire was based on the AASM criteria, widely recognized in sleep medicine.

## Results

A total of 300 participants (151 males, 149 females) aged 10–19 years were included in the final analysis. The mean age of the participants was **16.4 ± 1.7 years**, with no significant difference in age between males and females (*p* > 0.05). Demographic characteristics are summarized in Table 1.

**Table 1:** Participant demographics

Gender	Number of Participants	Age (Mean ± SD)
Male	151	16.5 ± 1.8
Female	149	16.3 ± 1.7

The prevalence of video game addiction was significantly higher among males (55%) compared to females (45%) (χ² = 4.12, *p* = 0.04), with an overall prevalence of 50.3% (*n* = 151). Among those with video game addiction, 70% (*n* = 106) reported high stress levels, while 30% (*n* = 45) reported low stress levels. In terms of gender, 60% of males (*n* = 91) and 50% of females (*n* = 75) exhibited high stress levels. *(See* Table 2*).*

**Table 2:** Prevalence of video game addiction, stress, and Bruxism by gender

Variable	Male	Female	*p*-value
**Video Game Addiction**	55% (= 83/151)	45% (= 67/149)	0.04
**High Stress Levels**	60% (= 91/151)	50% (= 75/149)	0.10
**Awake Bruxism**	55% (= 55/151)	50% (= 75/149)	0.25
**Sleep Bruxism**	45% (= 68/151)	40% (= 60/149)	0.35

**Table 3:** Correlation analysis between video game addiction and Bruxism

Variable	Bruxism Type	Correlation Coefficient (*r*)	*p*-value
Video Game Addiction	Awake Bruxism	0.31	< 0.001
Video Game Addiction	Sleep Bruxism	0.28	< 0.001

The mean Perceived Stress Scale (PSS) score for the entire sample was 28.4 ± 6.2. Participants with video game addiction had significantly higher stress levels (30.1 ± 5.8) compared to those without addiction (26.7 ± 6.5) (t = 4.56, *p* < 0.001). However, there were no significant gender differences in stress levels (*p* > 0.05). (See Table 2).

The overall prevalence of awake bruxism was 52.7% (*n* = 158), with a higher prevalence among participants with video game addiction (55%) compared to non-addicted participants (50%). Similarly, sleep bruxism was observed in 42.3% (*n* = 127) of the sample, with slightly higher rates among individuals with video game addiction (45%) compared to non-addicted individuals (40%). No significant gender differences were observed in the prevalence of either awake or sleep bruxism (*p* > 0.05). (See Table 2).

A statistically significant, albeit moderate, correlation was observed between video game addiction and bruxism (awake bruxism: *r* = 0.31, *p* < 0.001); sleep bruxism *r* = 0.28, *p* < 0.001), suggesting an association but not causality (See Table 3). Although bruxism was more common among individuals with high stress levels, the correlations between stress and both awake bruxism (*r* = 0.18, *p* = 0.12) and sleep bruxism (*r* = 0.20, *p* = 0.09) were not statistically significant.

Among participants with high stress levels (*n* = 180), 60% exhibited awake bruxism, while 50% exhibited sleep bruxism. In contrast, among those with low stress levels (*n* = 120), the prevalence of awake and sleep bruxism was 40% and 30%, respectively. Despite these differences, the association between stress and bruxism was not statistically significant (*p* > 0.05).

In summary, males were significantly more likely to exhibit video game addiction than females. Video game addiction was associated with higher stress levels and an increased likelihood of both awake and sleep bruxism. However, no significant correlation was found between stress levels and bruxism.

## Discussion

The findings of this study reveal a significant association between video game addiction and both awake and sleep bruxism in adolescents, while also highlighting the complex relationship between stress and bruxism. These results contribute to the growing body of literature on the psychological and physiological consequences of excessive video game use, particularly among adolescents, a population increasingly vulnerable to digital addiction.

The positive correlations observed between video game addiction and both awake (*r* = 0.31, *p* < 0.001) and sleep bruxism (*r* = 0.28, *p* < 0.001) suggest that excessive gaming may contribute to the development or increase in severityof bruxism. Kaess et al. found a positive correlation between video game addiction and stress reactivity [19]. The association between video game addiction and bruxism may be explained by increased neuromuscular activity during prolonged gaming sessions. However, additional research using objective electromyographic (EMG) assessments is needed to confirm this hypothesis.Similarly, Tinastepe and Iscan found a positive relationship between excessive smartphone use and sleep bruxism [20]. Both smartphone and video game addiction may cause constant tension in the jaw muscles due to activities that require continuous attention and are performed in long-term static positions. Additionally, individuals who play games for extended periods may be exposed to stressful gaming environments, increasing physiological stress responses and potentially triggering bruxism. This is consistent with previous research indicating that prolonged periods of gaming can lead to increased muscle tension, particularly in the jaw and facial muscles, due to the static postures and intense focus required during gameplay [21, 22]. Cioffi et al. demonstrated that individuals engaged in tasks requiring sustained attention, such as video gaming, exhibited increased jaw clenching behavior, which may explain the higher prevalence of bruxism in this population [21].

While video game addiction was associated with higher stress levels, the lack of a significant correlation between stress and bruxism in our study (awake: *r* = 0.18, *p* > 0.05; sleep: *r* = 0.20, *p* > 0.05) suggests that the relationship between these variables may be more nuanced than previously thought. This finding contrasts with some earlier studies that have identified stress as a key risk factor for bruxism [4, 5, 23]. For instance, Manfredini et al. found that psychosocial stress, particularly work-related stress, was a significant predictor of bruxism in adults [24]. However, our results indicate that in adolescents, other factors, such as gaming-related muscle tension or sleep disturbances, may play a more direct role in the development of bruxism.

It is also important to note that the assessment of bruxism in this study was limited to clenching and grinding behaviors, which may not fully capture the broader spectrum of awake bruxism manifestations. Bracing and thrusting behaviors, although recognized as part of the comprehensive definition of bruxism, were not evaluated and may contribute to underestimation.

Our study found that males were significantly more prone to video game addiction than females (55% vs. 45%, *p* < 0.05), which is consistent with previous research on gender differences in gaming behavior [ 25,26]. Desai et al. reported that males were more likely to engage in problematic gaming and experience associated health issues, such as poor sleep quality and psychological distress [8]. This gender disparity may be attributed to differences in gaming preferences, with males more likely to engage in competitive or action-oriented games that require prolonged periods of focused attention [25].

However, despite the higher prevalence of video game addiction in males, we found no significant gender differences in stress levels or bruxism prevalence. This suggests that while males may be more susceptible to video game addiction, the physiological and psychological consequences of addiction, such as bruxism and stress, may affect both genders equally. This finding contrasts with some studies that have reported higher rates of bruxism in females, possibly due to hormonal or psychosocial factors [9, 26]. Further research is needed to explore the role of gender in the relationship between video game addiction, stress, and bruxism.

The findings of this study have important implications for the health and well-being of adolescents. Video game addiction is increasingly recognized as a public health concern, with potential consequences ranging from psychological distress to physical health problems [27]. Our results suggest that bruxism may be an underrecognized consequence of excessive gaming, particularly in adolescents who are still developing coping mechanisms for stress and emotional regulation.

Additionally, stress management strategies, such as mindfulness or cognitive-behavioral therapy, may help mitigate the psychological and physiological effects of gaming addiction [28].

### Limitations and future directions

While this study provides valuable insights into the relationship between video game addiction, stress, and bruxism, several limitations should be acknowledged. First, the cross-sectional design precludes causal inferences, and longitudinal studies are needed to establish the temporal relationship between these variables. Second, the reliance on self-reported data may introduce bias, particularly in the assessment of bruxism and stress. Future studies could incorporate objective measures, such as polysomnography for sleep bruxism and salivary cortisol levels for stress [26, 29].

Additionally, the sample was limited to adolescents in a specific region, which may limit the generalizability of the findings. Future research should include more diverse populations and explore potential moderating factors, such as socioeconomic status, academic pressure, and family dynamics, that may influence the relationship between video game addiction and bruxism [30].

Additionally, the bruxism evaluation in this study did not include bracing and thrusting behaviors, which are part of the extended diagnostic criteria. This omission may limit the comprehensiveness of the bruxism assessment.

## Conclusion

In conclusion, this findings suggest a potential link between video game addiction and bruxism in adolescents, warranting further investigation through longitudinal and experimental studies. Preventive measures should focus on promoting healthier gaming habits and stress management strategies.Future research should explore the underlying mechanisms linking gaming, stress, and bruxism, as well as the long-term consequences of these behaviors on oral and overall health.

## Electronic supplementary material

Below is the link to the electronic supplementary material.

Supplementary Material 1

Supplementary Material 2

Supplementary Material 3

## Data Availability

The data supporting the findings of this study are available from the corresponding author upon reasonable request.

## Abbreviations

DGAS-21: Digital Game Addiction Scale-21
PSS: Perceived Stress Scale
AASM: American Academy of Sleep Medicine
TMJ: Temporomandibular Joint
EMG: electromyography
