# Supplementary material for: Association between video game addiction, stress, and bruxism in adolescents: a cross-sectional study
Source: BMC Oral Health. 2025 Jul 15;25:1164. doi: 10.1186/s12903-025-06568-0 (PMC12265209; doi:10.1186/s12903-025-06568-0)
Supplement: Supplementary file 1 — Supplementary Material 1 [file 12903_2025_6568_MOESM1_ESM.docx]

The original surveys were conducted in Turkish. For clarity and transparency, the original questionnaires in Turkish are provided below. An English translation is also provided for international readability.

Bruxism was evaluated using a self-report questionnaire consisting of 8 items organized into 3 sections based on the diagnostic criteria established by the American Academy of Sleep Medicine (AASM).

**Lütfen aşağıdaki soruları son 6 ayı düşünerek cevaplayınız.**

| 1. **Sizce uykunuzda sıklıkla dişlerinizi gıcırdatıyor musunuz? (evet/hayır)** |
| --- |
| 1. **Dişlerinizdeki aşınma olması gerekenden fazla mı? (evet/hayır)** |
| 1. **Uyanık olduğunuzda aşağıdaki semptomlardan herhangi birine sahip misiniz? (evet/hayır)** 2. **Uyanınca çenede yorgunluk, gerginlik ve ağrı hissi** 3. **Uyanınca dişlerinizi sıktığınızı ya da çenenizin ağrıdığı hissetme** 4. **Uyanınca şakaklarda ağrı** 5. **Uyanınca ağzı büyük açmada zorluk** 6. **Uyanınca çene ekleminde gerginlik hissi ve alt çeneyi serbestleştirmek için hareket ettirme gerekliliği** 7. **Uyanınca duyulan sonrasında kaybolan çene ekleminde klik sesi duyma** |

Participants answered the following questions considering the past 6 months:

**Section 1:**

- Do you think you frequently grind your teeth during sleep? (yes/no)

**Section 2:**

- Is the wear on your teeth greater than normal? (yes/no)

**Section 3:** (Associated Symptoms)

- Do you experience any of the following upon awakening? (yes/no)
  - Fatigue, tension, or pain in your jaw
  - Feeling of jaw clenching or jaw pain
  - Pain in the temples
  - Difficulty in opening your mouth wide
  - Jaw joint stiffness and need to move your lower jaw to relax
  - Clicking sound in the jaw joint that disappears later

For a positive diagnosis of sleep bruxism, participants had to answer "yes" to at least one question from Section 1 and/or Section 2 and additionally answer "yes" to at least one symptom from Section 3.

Awake bruxism was assessed with a single question asking whether participants had noticed themselves clenching or grinding their teeth during the day in the past 6 months (yes/no).

Only clenching and grinding behaviors were assessed; bracing and thrusting behaviors were not evaluated, which is explicitly acknowledged as a study limitation.
