# Supplementary material for: Association between video game addiction, stress, and bruxism in adolescents: a cross-sectional study
Source: BMC Oral Health. 2025 Jul 15;25:1164. doi: 10.1186/s12903-025-06568-0 (PMC12265209; doi:10.1186/s12903-025-06568-0)
Supplement: Supplementary file 2 — Supplementary Material 2 [file 12903_2025_6568_MOESM2_ESM.docx]

**Stress Levels (Perceived Stress Scale - PSS)**

Stress was assessed using the validated Turkish version of the Perceived Stress Scale (PSS), which measures the frequency of stress-related feelings and thoughts during the last month.

| 1. Geçen ay, beklenmedik bir şeylerin olması nedeniyle ne sıklıkta rahatsızlık duydunuz? |
| --- |
| 1. Geçen ay, hayatınızdaki önemli şeyleri kontrol edemediğinizi ne sıklıkta hissettiniz? |
| 1. Geçen ay, kendinizi ne sıklıkta sinirli ve stresli hissettiniz? |
| 1. Geçen ay, kişisel sorunlarınızı ele alma yeteneğinize ne sıklıkta güven duydunuz? |
| 1. Geçen ay, her şeyin yolunda gittiğini ne sıklıkta hissettiniz? |
| 1. Geçen ay, ne sıklıkta yapmanız gereken şeylerle başa çıkamadığınızı fark ettiniz? |
| 1. Geçen ay, hayatınızdaki zorlukları ne sıklıkta kontrol edebildiniz? |
| 1. Geçen ay, ne sıklıkta her şeyin üstesinden geldiğinizi hissettiniz? |
| 1. Geçen ay, ne sıklıkta kontrolünüz dışında gelişen olaylar yüzünden öfkelendiniz? |
| 1. Geçen ay, ne sıklıkta problemlerin üstesinden gelemeyeceğiniz kadar biriktiğini hissettiniz? |

The 10-item scale includes:

1. In the last month, how often have you been upset because of something unexpected?
2. In the last month, how often have you felt unable to control important things in your life?
3. In the last month, how often have you felt nervous and stressed?
4. In the last month, how often have you felt confident about your ability to handle personal problems?
5. In the last month, how often have you felt that things were going your way?
6. In the last month, how often have you found that you could not cope with all the things that you had to do?
7. In the last month, how often have you been able to control irritations in your life?
8. In the last month, how often have you felt that you were on top of things?
9. In the last month, how often have you been angered because of things that were outside your control?
10. In the last month, how often have you felt difficulties were piling up so high that you could not overcome them?

Responses were recorded on a 5-point Likert scale ranging from **"never (1)"** to **"always (5)"**. The scoring for negatively worded items was reversed. Total scores ranged from 10 to 50, with higher scores indicating higher levels of perceived stress.
