# Supplementary material for: Association between video game addiction, stress, and bruxism in adolescents: a cross-sectional study
Source: BMC Oral Health. 2025 Jul 15;25:1164. doi: 10.1186/s12903-025-06568-0 (PMC12265209; doi:10.1186/s12903-025-06568-0)
Supplement: Supplementary file 3 — Supplementary Material 3 [file 12903_2025_6568_MOESM3_ESM.docx]

**Digital Game Addiction Scale-21 (DGAS-21)**

Video game addiction was evaluated using the Turkish version of the short form of the Digital Game Addiction Scale-21 (DGAS-21) developed by Lemmens et al. The scale consists of 21 items, organized into 7 sub-dimensions.

| 1. Dijital oyunlar hayatımın olmazsa olmaz bir parçasıdır (benim içim çok önemlidir). |
| --- |
| 1. Dijital oyunun olmadığı bir hayat bana sıkıcı gelir. |
| 1. Dijital oyunun olmadığı bir hayat bana anlamsız gelir. |
| 1. Dijital oyun oynadığım için başka türlü eğlenceli aktivitelere (spor, müzik gibi) zamanım kalmaz. |
| 1. Sabah uyandığımda aklıma gelen ilk şey dijital oyun oynamak olur. |
| 1. Okul dışındaki vaktimin çoğunu dijital oyun oynayarak geçiririm. |
| 1. Okula gitmek yerine dijital oyun oynamayı tercih ederim. |
| 1. Bilgisayar, telefon, tablet ve konsol gibi dijital oyun araçlarından uzak kalmayı istemem. |
| 1. Başkaları ile yüz yüze sohbet etmek yerine dijital oyun oynamayı tercih ederim. |
| 1. Dijital oyun oynarken tuvalet ihtiyacımı ertelediğim zamanlar olur. |
| 1. İstediğim zaman dijital oyun oynayamazsam sinirlenirim/öfkelenirim. |
| 1. Dijital oyun oynamadığım zaman iştahım kaçar. |
| 1. Dijital oyun oynamadığım zaman kendimi huzursuz hissederim. |
| 1. Sınıfta ders esnasında dijital oyun oynamayı hayal ederim. |
| 1. Ev dışında herhangi bir yere gittiğimde dijital oyun oynayabileceğim bir araç (bilgisayar, telefon, tablet, konsol vb.) var mı diye etrafa bakınırım. |
| 1. Bilgisayar, cep telefonu, tablet gibi teknolojik araçları gördüğümde aklıma gelen ilk şey dijital oyun oynamak olur. |
| 1. Mutsuz olduğum zamanlarda dijital oyun oynamak beni rahatlatır. |
| 1. Her defasında daha uzun süre dijital oyun oynamak isterim. |
| 1. Dijital oyun oynarken acıktığımın farkına varmam. |
| 1. Gün içerisinde birdenbire/aniden dijital oyun oynamayı istediğim zamanlar olur. |
| 1. Ailemle vakit geçirmek yerine dijital oyun oynamayı tercih ederim. |

Participants rate each statement on a 5-point Likert scale:
(1 = Never, 2 = Rarely, 3 = Sometimes, 4 = Frequently, 5 = Always)

1. Digital games are an essential part of my life (very important for me).
2. Life without digital games seems boring to me.
3. Life without digital games seems meaningless to me.
4. Due to playing digital games, I don't have time for other enjoyable activities (such as sports or music).
5. When I wake up in the morning, the first thing that comes to my mind is playing digital games.
6. I spend most of my time outside school playing digital games.
7. I prefer playing digital games instead of going to school.
8. I don't want to stay away from digital gaming devices such as computers, phones, tablets, or consoles.
9. I prefer playing digital games instead of having face-to-face conversations with others.
10. Sometimes I delay going to the toilet because I’m playing digital games.
11. I get angry or annoyed if I cannot play digital games whenever I want.
12. When I'm not playing digital games, I lose my appetite.
13. When I'm not playing digital games, I feel restless.
14. During class, I daydream about playing digital games.
15. When I'm away from home, I check if there is a device around (like a computer, phone, tablet, console, etc.) on which I could play digital games.
16. When I see technological devices such as computers, mobile phones, or tablets, the first thing that comes to mind is playing digital games.
17. Playing digital games helps me relax when I am unhappy.
18. Each time, I feel the desire to play digital games for a longer period.
19. When I'm playing digital games, I don't realize I'm hungry.
20. Sometimes during the day, I suddenly have an urge to play digital games.
21. I prefer playing digital games instead of spending time with my family.
